# DTI‐ALPS as a Biomarker of Small Vessel Disease Progression and Amyloid‐β in Normal Aging: A 3‐Year Longitudinal Study

**DOI:** 10.1002/hbm.70649

**Published:** 2026-09-20

**Authors:** Narges Azizi, Hoda Borooghani, Iman Kiani, Zeinab Gharaylou, Dina Seyedi, Kavous Firouznia, Shahriar Kolahi

**Affiliations:** ^1^ Advanced Diagnostic and Interventional Radiology Research Center Tehran University of Medical Sciences Tehran Iran; ^2^ Tehran University of Medical Sciences Tehran Iran

**Keywords:** amyloid PET, cerebral small vessel disease, cognition, DTI‐ALPS, glymphatic system, white matter hyperintensities

## Abstract

Cerebral small vessel disease (SVD) is a leading cause of stroke, vascular cognitive impairment, and functional decline in older adults. Emerging experimental and translational data implicate glymphatic dysfunction in SVD pathogenesis and in vascular amyloid accumulation. The analysis along the perivascular space (ALPS) index, derived from diffusion tensor imaging (DTI), noninvasively estimates water diffusivity along perivascular pathways and has been proposed as an indirect imaging marker related to glymphatic function. In this study, we aim to determine whether the baseline DTI‐ALPS index is associated with baseline SVD burden and subsequent longitudinal changes in SVD markers and amyloid‐β deposition in cognitively normal aging. We analyzed 204 cognitively normal participants from the Harvard Aging Brain Study with baseline and follow‐up visits separated by 3 years. Primary MRI SVD outcomes were intracranial‐volume–normalized white matter hyperintensities (WMH/ICV), and visual SVD ratings (Fazekas, ARWMC, perivascular space, and BOMBS [microbleed scale]). Secondary outcomes included cortical amyloid PET burden (PIB_FS_DVR_FLR). We tested the effects of time, baseline ALPS (*z*‐scored), and the time‐ALPS interaction using Bayesian mixed‐effects models with subject‐specific random intercepts adjusted for baseline age, sex, and education; amyloid models were additionally adjusted for APOE4 status. Continuous outcomes were modeled with Gaussian mixed‐effects models, binary outcomes with Bernoulli mixed‐effects models, and ordinal outcomes with Bayesian cumulative mixed‐effects models. In a sensitivity analysis, ALPS was recalculated after excluding voxels with *λ*_2_/*λ*_3_ > 1.8, and the models were repeated using the crossing‐fiber‐adjusted index. Posterior means and 95% HDIs were the primary summaries. Multiplicity was controlled using the Benjamini–Hochberg false discovery rate procedure, and the resulting FDR‐adjusted two‐sided posterior tail‐area *p*‐analogues are reported as *p*_corrected_. Over the 3‐year follow‐up, SVD and amyloid‐β burden increased: WMH/ICV (*β* = 0.096, 95% HDI [0.069, 0.121], *p*_corrected_ < 0.001), cortical amyloid‐β (*β* = 0.130, 95% HDI [0.106, 0.152], *p*_corrected_ < 0.001), Fazekas (*β* = 0.320, 95% HDI [0.088, 0.541], *p*_corrected_ = 0.010), ARWMC (*β* = 0.404, 95% HDI [0.165, 0.649], *p*_corrected_ = 0.004), basal ganglia (*β* = 0.318, 95% HDI [0.079, 0.546], *p*_corrected_ = 0.010), and centrum semiovale (*β* = 0.244, 95% HDI [0.026, 0.450], *p*_corrected_ = 0.025). PVS scores also increased over time. Higher baseline ALPS was associated with lower Fazekas severity (*β* = −0.742, 95% HDI [−1.281, −0.201], *p*_corrected_ = 0.026) and with attenuated amyloid accumulation over time (*β* = −0.025, 95% HDI [−0.049, −0.001], *p*_corrected_ = 0.039). In a sensitivity analysis excluding voxels with a high likelihood of substantial fiber crossing, the ALPS‐by‐time interaction for amyloid burden remained significant (*β* = −0.028, 95% HDI [−0.052, −0.003], *p*_corrected_ = 0.022), whereas the baseline association with Fazekas severity was attenuated (*β* = −0.413, 95% HDI [−0.966, 0.181], *p*_corrected_ = 0.751). Higher baseline WMH/ICV was associated with poorer baseline global cognition (PACC5) (*β* = −0.092, 95% HDI [−0.177, −0.011], *p*_corrected_ = 0.036), processing speed (*β* = −0.116, 95% HDI [−0.223, −0.010], *p*_corrected_ = 0.036), and executive function (*β* = −0.144, 95% HDI [−0.251, −0.029], *p*_corrected_ = 0.035). Higher baseline amyloid burden was associated with steeper PACC5 decline (*β* = −0.065, 95% HDI [−0.096, −0.033], *p*_corrected_ < 0.001). In cognitively normal older adults, higher conventional baseline ALPS was associated with lower Fazekas severity, although attenuation after crossing‐fiber adjustment indicates that this cross‐sectional relationship was sensitive to the microstructural composition of the ALPS regions. In contrast, higher baseline ALPS was associated with slower short‐term amyloid‐β accumulation, and this longitudinal association remained significant after crossing‐fiber adjustment. Greater baseline amyloid burden was associated with more rapid decline in global cognition, whereas higher baseline WMH volume was associated with poorer baseline global cognition, processing speed, and executive function. These findings support ALPS as a candidate noninvasive diffusion marker associated with longitudinal amyloid‐related change, while emphasizing the need for further validation of its relationship with SVD burden and its biological specificity.

## Key Points

- **DTI‐ALPS as an SVD marker**: Higher baseline ALPS index was cross‐sectionally associated with a lower Fazekas score in cognitively normal older adults.
- **DTI‐ALPS and amyloid progression**: Higher baseline ALPS was linked to attenuated longitudinal amyloid‐β accumulation.
- **Clinical relevance of amyloid and white matter hyperintensity volume**: Amyloid burden is associated with steeper cognitive decline, while higher white matter hyperintensity volume is associated with poorer baseline global, processing, and executive function.

## Graphical abstract

A higher DTI‐derived ALPS index relates cross‐sectionally to both quantitative and visual measures of SVD and is associated with short‐term amyloid‐β accumulation in cognitively normal older adults. Also, greater amyloid burden and choroid plexus volume are associated with a more rapid deterioration in global cognition and processing speed.

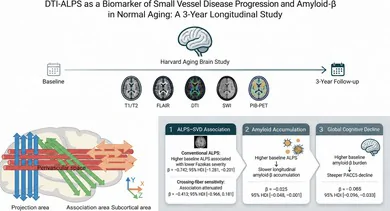

## 1 Introduction

Cerebral small vessel disease (SVD) represents one of the most common and clinically important causes of stroke, vascular cognitive impairment, and functional decline in aging populations (Wardlaw et al. 2019). Pathologically, SVD involves damage to the perforating arterioles, capillaries, and venules of the brain, leading to a spectrum of parenchymal lesions visible on MRI, including white matter hyperintensities (WMH), lacunes, cerebral microbleeds, and enlarged perivascular spaces (PVS) (Gouw et al. 2011; Passiak et al. 2019). These features collectively form the total SVD burden, which correlates strongly with cognitive deterioration, gait disturbance, and risk of recurrent cerebrovascular events (Østergaard et al. 2016). Despite substantial advances in structural neuroimaging, the mechanisms driving SVD progression remain incompletely understood, and current imaging markers largely reflect established tissue injury rather than early microvascular dysfunction.

Emerging evidence points to a role for glymphatic dysfunction, the failure of cerebrospinal fluid (CSF)–interstitial fluid exchange, in the pathogenesis of SVD (Ang et al. 2024). The glymphatic system, facilitated by perivascular channels lined with aquaporin‐4 (AQP4) water channels on astrocytic end‐feet, mediates waste clearance, and interstitial solute transport throughout the brain (Rasmussen et al. 2018). Impairment of this system may lead to metabolic waste accumulation, blood–brain barrier disruption, and secondary vascular and white matter injury. Animal studies have shown that reduced glymphatic function is associated with arteriolosclerotic small‐vessel disease, white matter damage, and vascular amyloid deposition in amyloid‐related forms of SVD (Mestre et al. 2017). Translating these findings to human studies, however, has been challenging due to the lack of noninvasive imaging biomarkers that can quantify glymphatic activity in vivo.

The analysis along the perivascular space (ALPS) index, derived from diffusion tensor imaging (DTI), has been proposed as a noninvasive indirect marker related to glymphatic function in the human brain (Taoka et al. 2017). It estimates water diffusivity along perivascular pathways at the level of the medullary veins, where diffusion is directionally aligned with perivascular channels. Recent studies have shown that the ALPS index decreases with aging and correlates with amyloid burden, sleep disruption, and neurodegenerative pathology (Voorter et al. 2024; Hsiao et al. 2023; Kamagata et al. 2022; Siow et al. 2022).

However, the role of the ALPS index as a marker of longitudinal SVD progression remains unestablished. Most existing studies have been cross‐sectional, limiting the ability to infer temporal relationships. Establishing whether the ALPS index is associated with subsequent accumulation of SVD lesions could provide a valuable early marker for disease risk and progression, offering opportunities for preventive intervention before irreversible damage occurs. In this study, we aimed to determine whether the baseline ALPS index is associated with 3‐year SVD burden, including WMH volume, lacunes, PVS grade, and cerebral microbleeds, in cognitively normal older adults. In addition, we explored the relationship between cognitive performance and imaging markers related to glymphatic and perivascular dynamics, including the ALPS index, choroid plexus (CP) volume, basal ganglia perivascular space (BG‐PVS) burden, and cerebral amyloid deposition, to clarify their contributions to cognitive aging in the preclinical SVD spectrum.

## 2 Materials and Methods

### 2.1 Study Population

**FIGURE 1:**
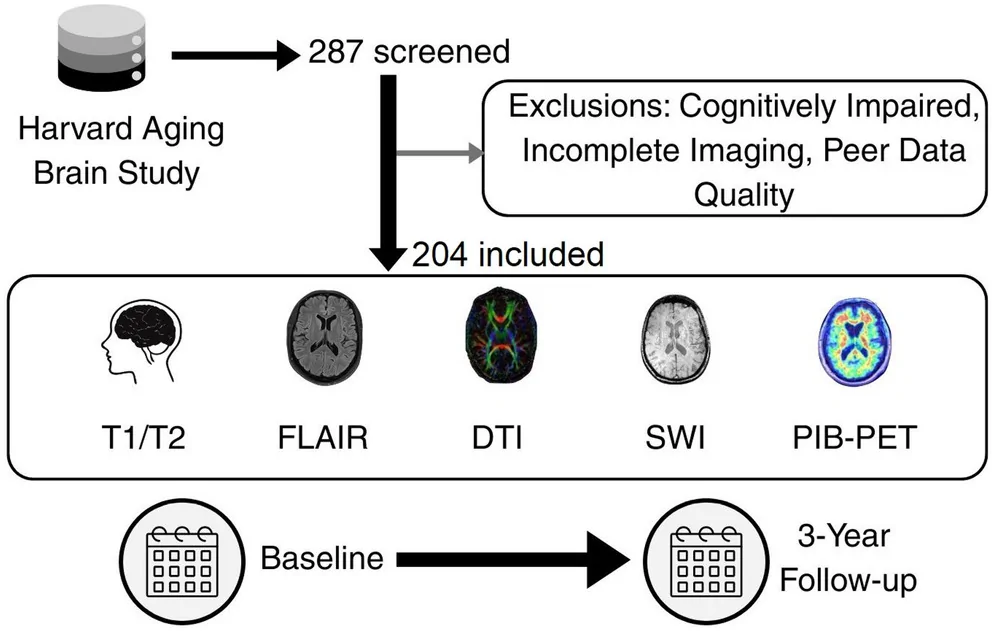
An overview of the study cohort and imaging data.

Participants were drawn from the Harvard Aging Brain Study (HABS), a longitudinal cohort to investigate brain aging using multimodal neuroimaging and neuropsychological assessments in older adults (Dagley et al. 2017). For the present analysis, inclusion criteria required participants to be older than 60 years at baseline and to meet the HABS criteria for cognitively normal status (Clinical Dementia Rating [CDR] global score of 0, Mini–Mental State Examination [MMSE] score > 25, performance above age‐ and education‐adjusted cutoffs on the 30‐minute delayed recall of Logical Memory Story A, and Geriatric Depression Scale [GDS] score < 11). Participants were also required to have diffusion MRI for calculation of ALPS index, high‐resolution structural MRI for assessment of SVD markers, and longitudinal data from at least two study visits, including baseline (HAB_1.0) and approximately 3‐year follow‐up (HAB_4.0). Individuals with a diagnosis of Alzheimer's disease or mild cognitive impairment, or with missing neuroimaging data at either the baseline or 3‐year follow‐up visit, were excluded. In addition, individuals with head trauma, substance abuse, alcoholism, or serious medical or psychiatric illness were excluded per HABS protocol. All participants provided written informed consent, and the study protocol was approved by the local institutional review board in accordance with the Declaration of Helsinki. An overview of the study data is presented in Figure 1.

### 2.2 Neuropsychological Assessment

Participants underwent the neuropsychological assessment battery at each study visit to evaluate cognitive domains, including global cognition, episodic memory, executive function, attention and working memory, language, processing speed, and visuospatial abilities. The battery included measures of confrontation naming (Boston Naming Test), category fluency (animals, vegetables, and fruits), phonemic verbal fluency (FAS total and letter‐specific scores), digit span (forward and backward), Digit Symbol Substitution Test, Trail Making Test parts A and B, Logical Memory immediate and delayed recall, the Free and Cued Selective Reminding Test (FCSRT; free recall and total recall with cueing), and the Selective Reminding Test (total recall, delayed recall, consistent long‐term retrieval, long‐term storage, short‐term recall, and intrusions). Additional task‐specific performance indices, including perseverations, intrusions, commissions, omissions, sequencing errors, and set‐shifting errors (for category fluency, FAS, TMT‐A, and TMT‐B), were retained. Global cognitive status was assessed using the MMSE, with domain‐specific subscores for orientation, immediate recall, attention/calculation, delayed recall, language, and visuoconstruction. Functional and clinical status were also characterized using the CDR, and depressive symptoms were assessed using the GDS.

A composite measure of global cognition was derived using the Preclinical Alzheimer Cognitive Composite 5 (PACC5), including: (1) Free and Cued Selective Reminding Test total recall (FCSRT96; range 0–96), (2) delayed recall from the Logical Memory IIa subtest of the Wechsler Memory Scale (0–25), (3) Digit Symbol Substitution Test score (0–93), (4) MMSE total score (0–30), and (5) CAT3 (sum of animals, vegetables, and fruits) (Papp et al. 2022, 2017). For each component, raw scores for each component were z‐transformed at each time point using the mean and standard deviation of the study sample at baseline. The PACC5 composite score was then calculated as the arithmetic mean of these five standardized *z*‐scores for each participant.

Processing speed was quantified using a validated proxy composite derived from the Digit Symbol Substitution Test and TMT‐A (Hoogendam et al. 2014):

$${\text{Processing Speed}}_{z}=\frac{z\left(\text{Digit Symbol}\right)-z\left(\mathrm{TMT}-A\right)}{2}$$

Executive function was quantified using a Trail Making Test–based proxy defined as the difference between Part B and Part A completion times in seconds (TMT‐B—TMT‐A) (Sánchez‐Cubillo et al. 2009). Because higher raw difference scores indicate poorer performance, this measure was standardized using the baseline mean and standard deviation of the study sample and sign‐inverted so that higher values reflect better executive function:

$${\text{Executive Function}}_{z}=-z\left[\left(\mathrm{TMT}-B\right)-\left(\mathrm{TMT}-A\right)\right]$$

### 2.3 MRI Acquisition and Image Processing

High‐resolution T1‐weighted structural magnetic resonance images were acquired as part of the HABS neuroimaging protocol. Images were obtained using a three‐dimensional T1‐weighted sequence with whole‐brain coverage (matrix size: 240 × 256 × 176), with a spatial resolution of 1.05 × 1.05 × 1.20 mm^3^. Cortical and subcortical segmentation, surface reconstruction, and cortical thickness estimation were performed using FreeSurfer (version 7.3.2). Processing included skull stripping, intensity non‐uniformity correction, automated tissue classification, topology correction, and generation of subject‐specific cortical surface models. CP segmentation was refined using a previously published Gaussian Mixture Model (GMM)‐based method (Tadayon et al. 2020). A quality‐control subset of 126 scans from 63 participants underwent expert review of the bilateral CP masks by a neuroradiologist with 5 years of experience, blinded to ALPS, amyloid, SVD, and cognitive measures. Masks were manually corrected for nonanatomical extensions, inclusion of ventricular CSF or adjacent structures, inaccurate CP–CSF boundaries, and omission of identifiable CP tissue. Any voxel‐level modification was classified as a correction. Automated‐to‐expert agreement was quantified using Dice coefficients.

The PVS segmentation process was performed using the PINGU pipeline, which is designed to extract PVS in brain regions from T1‐weighted MRI scans (Sinclair et al. 2026). To assess the basal ganglia PVS, masks were created on a case‐by‐case basis using FreeSurfer's mri_binarize, applied to the aseg.mgz to generate binary masks of the basal ganglia. A 2 mm exclusion zone was applied around the ventricles to eliminate PVS voxels close to the ventricles for final analysis.

T2‐weighted fluid‐attenuated inversion recovery (FLAIR) images were acquired with a matrix size of 256 × 256 × 120 and a spatial resolution of 1.0 × 1.0 × 1.5 mm^3^. FLAIR images underwent visual quality control and were coregistered to each participant's T1‐weighted structural MRI. These images were used to identify white‐matter hyperintensities in secondary analyses. To quantify the burden of WMH, the UBO Detector pipeline was employed (Jiang et al. 2018). All outputs were visually inspected, and volume was normalized by the individual's intracranial volume (ICV) to account for differences in brain size.

DTI data were acquired using a single‐shot echo‐planar imaging sequence with 35 volumes, including 5 non‐diffusion‐weighted images and 30 diffusion‐weighted directions. Images were acquired with isotropic voxel dimensions of 2 × 2 × 2 mm^3^, *a b*‐value of 700 s/mm^2^, TR/TE = 8040/84 ms, phase‐encoding direction j‐, and a total readout time of 0.05 s. The data were preprocessed using the DTI‐ALPS pipeline (https://github.com/gbarisano/alps/), which included denoising, unringing, eddy current distortion correction, and motion correction, followed by diffusion tensor fitting (Liu et al. 2024). For the calculation of the DTI‐ALPS index, the diffusion tensor was used to extract diffusivity maps along the perivascular space (Taoka et al. 2017). Registration was performed to align the diffusion tensor maps to the JHU‐ICBM‐FA‐1 mm template space. Following the automated FSL‐based procedure described by Liu et al., bilateral spherical ROIs with a radius of 2.5 mm were automatically placed within the superior corona radiata (projection fibers) and superior longitudinal fasciculus (association fibers) (Liu et al. 2024). The ALPS index was computed separately for each hemisphere, and the final value was obtained by averaging the left and right hemisphere indices.

To assess whether the primary ALPS findings were influenced by complex fiber architecture within the ROIs, we performed a sensitivity analysis using the eigenvalue‐ratio approach described by Georgiopoulos et al. (Georgiopoulos et al. 2024). For each participant, the ratio of the middle to the lowest diffusion‐tensor eigenvalue (*λ*_2_/*λ*_3_) was calculated in native diffusion space. Voxels with *λ*_2_/*λ*_3_ > 1.8, indicating a high likelihood of substantial fiber crossing, were excluded. The resulting binary retained‐voxel mask was transformed to template space using the same native‐to‐template transformation as the diffusion maps with nearest‐neighbor interpolation and intersected with the four ALPS ROIs. Mean D_xx_, D_yy_, and D_zz_ were recalculated within the retained voxels, and the ALPS index was recomputed separately for each hemisphere and then averaged across hemispheres. All primary SVD, amyloid, and cognitive models were repeated using this crossing‐fiber‐adjusted baseline ALPS index.

Susceptibility‐weighted imaging (SWI) was acquired to assess microbleeds. SWI data consisted of a single three‐dimensional volume with a matrix size of 192 × 256 × 72, with a spatial resolution of 0.9 × 0.9 × 1.5 mm^3^.

Amyloid‐β deposition in the brain was assessed using positron emission tomography with 11C‐Pittsburgh Compound‐B (PiB PET). PET imaging was conducted using a Siemens ECAT HR+ scanner. Dynamic PiB PET data were acquired over a 60‐min period post‐injection, with 39 temporal frames and a spatial resolution of approximately 2.1 × 2.1 × 2.4 mm^3^. The dynamic PET frames were corrected for motion and coregistered to each participant's corresponding T1‐weighted MRI. Partial volume correction was applied using the geometric transfer matrix method to mitigate spill‐in and spill‐out effects due to regional atrophy. Distribution volume ratios (DVRs) were computed relative to uptake in the whole cerebellum for each subject. Global amyloid burden (PIB_FS_DVR_FLR) was calculated as the mean DVR across a FreeSurfer‐defined cortical composite region (FLR), comprising the frontal, lateral temporal and parietal, and retrosplenial cortices.

### 2.4 MRI Small Vessel Disease (SVD)

Small vessel disease (SVD) in this study was assessed using a combination of radiologic scoring and automated segmentation methods. Radiological assessment was conducted based on the Fazekas Scale for WMH from 0 (no hyperintensities) to 3 (severe WMH) (Duering et al. 2023). The Age‐Related White Matter Changes (ARWMC) scale was also applied to evaluate WMH in the periventricular and deep white matter regions, with a range from 0 (no changes) to 3 or 4 (severe involvement) (Duering et al. 2023). Additionally, PVS was assessed in the basal ganglia, centrum semiovale, and midbrain using radiologist‐determined scores. These PVS assessments range from 0 (no PVS) to 4 (severe involvement). The presence of lacunes was also recorded, with a binary score indicating presence or absence. The MARS scale was also applied to assess microangiopathy in infratentorial, deep, and lobar brain regions (Ringelstein and Nabavi 2005). Lastly, the BOMBS scale was used to evaluate lobar regions, basal ganglia, and posterior fossa lesions, with scores ranging from 0 (no involvement) to 3 (severe involvement) (Duering et al. 2023). In parallel, WMH were analyzed using the UBO study pipeline, and PVS segmentation was performed using the PINGU pipeline. The resulting maps were visually checked and minimally edited under the supervision of a neuroradiologist to ensure accuracy.

### 2.5 Statistical Analysis

The statistical analysis was performed to examine the association between the diffusion tensor imaging‐based ALPS index and the progression of small vessel disease (SVD) over a 3‐year follow‐up period. The primary predictor, ALPS_baseline_z, was derived by standardizing the baseline ALPS scores across participants and was used to assess its association with changes in SVD severity over time. All analyses were conducted using Python 3.13.2, pandas 2.2.3, NumPy 2.2.3, PyMC 5.27.0, Bambi 0.16.0, and ArviZ 0.23.0.

For continuous outcomes, including WMH/ICV and PIB_FS_DVR_FLR, Bayesian Gaussian mixed‐effects models were used to explore the relationship with baseline ALPS and longitudinal changes. For ordinal outcomes, including the Fazekas total score, ARWMC total score, and PVS ratings (for basal ganglia and centrum semiovale), Bayesian cumulative mixed‐effects models were used, incorporating random intercepts for subject variability. For binary outcomes, including MARS (for deep white matter, infratentorial, and lobar areas), BOMBS (for basal ganglia, posterior fossa, and lobar areas), PVS (for midbrain), and lacune presence, Bayesian Bernoulli mixed‐effects models were applied to assess their relationship with baseline ALPS and longitudinal changes. Posterior summaries are reported as point estimates with 95% highest density intervals (HDIs).

For each cognitive outcome (PACC5, processing speed, and executive function), a Bayesian Gaussian mixed‐effects model with subject‐specific random intercepts was used. The model included TimeYears (*z*‐score), baseline ALPS (*z*‐score), and their interaction (TimeYears_*z* × ALPS_baseline_*z*), as well as parallel baseline predictors including CP/ICV, WMH/ICV, BG‐PVS/ICV, and amyloid burden (PIB FS DVR FLR), together with the interaction of each baseline imaging measure with time. The cognitive model was repeated using expert‐corrected CP/ICV as a sensitivity analysis.

Age, gender, and years of education were included as covariates to adjust for potential confounders. In amyloid models, APOE4 status was additionally included as a covariate. Missing data were addressed using a model‐wise complete‐case approach, as the percentage of missing data for all variables was below 5%. No rows were globally dropped. Outliers in the outcomes were managed through winsorization within each session (baseline and follow‐up), with values outside the 1st and 99th percentiles capped at these thresholds. For volume normalization, ICV‐based normalization was applied to outcomes including BG‐PVS/ICV, WMH/ICV, and CP/ICV.

Gaussian imaging and amyloid models used Normal(0, 2) priors for regression coefficients, whereas cognitive models used Normal(0, 1.5) priors. In both model types, subject offsets were assigned Normal(0, 1) priors, and the random‐intercept and residual standard deviations were assigned Half‐Normal(1) priors. Bernoulli and cumulative‐logit models used Bambi's weakly informative autoscaled priors, with the full model‐specific specifications reported in Table S3. Posterior sampling used the No‐U‐Turn Sampler with four chains and a fixed seed of 42. Sampling length was selected by model class to ensure stable posterior estimation: continuous imaging and amyloid models used 3000 tuning iterations and 4000 retained draws per chain, whereas all other models used 2000 of each. Target acceptance was 0.95, except for cumulative‐logit models (0.93). Convergence was considered acceptable when rank‐normalized split‐$\hat{R}$ was ≤ 1.01, bulk and tail effective sample sizes were ≥ 400, no divergent transitions occurred, and the Bayesian fraction of missing information (BFMI) was ≥ 0.30. All primary models met these criteria; model‐specific diagnostics are reported in Table S4.

Posterior means and 95% HDIs were the primary summaries. The two‐sided posterior tail‐area *p*‐analogue was calculated as *p*_post = 2 × min[Pr(*β* > 0 | data), Pr(*β* < 0 | data)] and was interpreted descriptively rather than as a frequentist *p*‐value or the posterior probability of the null hypothesis.

The Benjamini–Hochberg procedure was applied to these posterior *p*‐analogues as a conservative multiplicity screen, separately for each model term within four prespecified families: WMH/ICV (*m* = 1), amyloid burden (*m* = 1), visual SVD measures (*m* = 5), and binary SVD measures (*m* = 7). Cognitive analyses were corrected across the three cognitive outcomes separately for each model term. *p*_corrected_ represents the Benjamini–Hochberg FDR‐adjusted two‐sided posterior tail‐area *p*‐analogue. It was interpreted as a supplementary multiplicity safeguard rather than as a frequentist *p*‐value or the posterior probability of the null hypothesis.

## 3 Results

The initial sample consisted of 287 patients from the HABS study. After excluding participants diagnosed with Alzheimer's disease and mild cognitive impairment, as well as those with incomplete structural and DTI data for both baseline and 3‐year follow‐up, 204 participants were included in the final analysis. The mean age of participants at baseline was 73.35 years (SD = 6.10), and the mean age at follow‐up was 76.42 years (SD = 6.08). Of the participants, 118 (58.4%) were female and 86 (41.6%) were male. Baseline demographic, cognitive, and neuroimaging data are summarized in the Table 1.

**TABLE 1:** Baseline participant characteristics and imaging markers.

	Baseline (*n* = 204)
Demographic features	
Age, year, mean ± SD (range)	73.35 ± 6.10 (62.50, 89.25)
Sex, *n* (%)	
Female	118 (57.84)
Male	86 (42.16)
Years of education, mean ± SD (Range)	16.08 ± 3.01 (6, 20)
APOE ϵ4 status, *n* (%)	
Negative (ϵ4−)	143 (70.10)
Positive (ϵ4+)	56 (27.45)
Missing	5 (2.45)
Cognitive tests	
FCSRT96, median [IQR] (range)	82.00 [7.00] (63.00, 96.00)
LMDR, median [IQR] (range)	14.00 [4.00] (6.00, 25.00)
Digit symbol, mean ± SD (range)	48.57 ± 9.98 (23.00, 86.00)
MMSE, median [IQR] (Range)	29.00 [1.00] (25.00, 30.00)
CAT, median [IQR] (range)	45.00 [13.00] (23.00, 79.00)
TMT‐A [IQR] (range)	32.86 [14.13] (11.00, 141.78)
TMT‐B [IQR] (range)	70.86 [39.08] (3.15, 300.00)
PACC5, mean ± SD (range)	0.00 ± 0.64 (−1.97, 2.07)
Processing speed_z_, mean ± SD (Range)	0.03 ± 0.80 (−2.77, 2.38)
Executive function_z_	0.34 [0.74] (−5.21, 2.44)
Imaging markers	
DTI‐ALPS, mean ± SD	1.23 ± 0.13
ICV (mL), median [IQR]	1455.38 [238.698]
CP Volume, mm^3^, mean ± SD	2723.72 ± 901.99
WMH, mL, median [IQR]	10.37 [11.03]
BG‐PVS, mL, median [IQR]	0.14 [0.12]
Amyloid‐β (PIB‐FS‐DVR‐FLR), median [IQR]	1.10 [0.15]

*Note:* SD, standard deviation; IQR, interquartile range; PACC5, Preclinical Alzheimer Cognitive Composite 5, computed as the mean of five cognitive component scores: Free and Cued Selective Reminding Test total recall, 0–96 scale (FCSRT96), delayed recall from the Logical Memory IIa subtest of the Wechsler Memory Scale (LMDR), Digit Symbol Substitution Test score (Digit Symbol), Mini‐Mental State Examination total score (MMSE), and Category Fluency total score (CAT; sum of animals, vegetables, and fruits); Processing speed *z*‐score: [z(Digit Symbol)‐z(TMT‐A)]/2; TMT‐A, Trail Making Test Part A; Executive function *z*‐score, −z[(TMT‐B)—(TMT‐A)]; TMT‐B, Trail Making Test Part B; APOE, apolipoprotein E; DTI‐ALPS, diffusion tensor imaging–analysis along the perivascular space; ICV, intracranial volume; CP, choroid plexus; WMH, white matter hyperintensity; BG‐PVS, basal ganglia perivascular spaces; PIB_FS_DVR_FLR, mean [11C] Pittsburgh Compound‐B (PIB) distribution volume ratio (DVR), referenced to whole‐cerebellum uptake, across a FreeSurfer‐defined cortical composite comprising the frontal, lateral temporal, parietal, and retrosplenial cortices.

Table 2 presents the visual ratings of SVD markers at baseline and at the 3‐year follow‐up. For WMH burden, the distribution of Fazekas scores showed a trend toward higher grades over time, with the proportion of participants with Fazekas grade 0 decreasing from 16.3% to 9.9%, while grades 2 and 3 increased from 29.7% to 33.7% and from 7.9% to 8.9%, respectively; however, this shift did not reach statistical significance (*p* = 0.278). Similarly, ARWMC scores demonstrated a non‐significant trend toward progression, with Grade 0 decreasing from 22.3% to 16.8% and Grade 2 increasing from 18.8% to 25.7% (*p* = 0.264). Perivascular space ratings remained stable across all anatomical regions. In the basal ganglia, the distribution of PVS grades was unchanged between baseline and follow‐up (*p* = 0.551), with Grade 2 remaining the most prevalent category (45.5% vs. 46.1%). Centrum semiovale PVS ratings were similarly stable (*p* = 0.644), as were midbrain PVS ratings (49.0% vs. 52.5%; *p* = 0.486) (Table 2).

**TABLE 2:** Visual small vessel disease (SVD) markers at baseline and 3‐year follow‐up.

Visual SVD score	Baseline	3‐Year follow‐up	*p*
Fazekas score, *n* (%)			0.278
0	33 (16.3)	20 (9.9)	
1	93 (46.0)	96 (47.5)	
2	60 (29.7)	68 (33.7)	
3	16 (7.9)	18 (8.9)	
ARWMC score, *n* (%)			0.264
0	45 (22.3)	34 (16.8)	
1	106 (52.5)	101 (50.0)	
2	38 (18.8)	52 (25.7)	
3	13 (6.4)	15 (7.4)	
Perivascular space (Basal Ganglia), *n* (%)			0.551
0	19 (9.4)	11 (5.4)	
1	60 (29.7)	59 (29.2)	
2	92 (45.5)	94 (46.1)	
3	30 (14.9)	36 (17.6)	
4	1 (0.5)	2 (1.0)	
Perivascular space (Centrum Semiovale), *n* (%)			0.644
0	21 (10.4)	20 (9.9)	
1	41 (20.3)	35 (17.3)	
2	66 (32.7)	67 (33.2)	
3	68 (33.7)	68 (33.7)	
4	6 (3)	12 (5.9)	
Perivascular space (Midbrain), *n* (%)	99 (49.0)	107 (52.5)	0.486
Lacune presence, *n* (%)	21 (10.4)	29 (14.4)	0.290
MARS, infratentorial, *n*(%)	7 (3.5)	9 (4.4)	0.621
MARS, deep white matter, *n* (%)	32 (15.8)	38 (18.8)	0.511
MARS, Lobar, *n* (%)	22 (10.9)	28 (13.9)	0.450
BOMBS, Lobar, *n* (%)	28 (13.7)	38 (18.8)	0.226
BOMBS, Basal Ganglia, *n* (%)	9 (4.5)	12 (6.0)	0.512
BOMBS, Posterior Fossa, *n* (%)	7 (3.4)	8 (3.9)	1.000

Abbreviations: ARWMC, age‐related white matter changes; BOMBS, Brain Observer MicroBleed Scale; MARS, Microbleed Anatomical Rating Scale; SVD, small vessel disease.

The prevalence of lacunes increased from 10.4% to 14.4%, although this difference was not statistically significant (*p* = 0.290). Cerebral microbleed prevalence, assessed using both MARS and BOMBS rating scales, remained stable across all regions. MARS‐rated microbleeds showed non‐significant increases in infratentorial (3.5%–4.4%; *p* = 0.621), deep white matter (15.8%–18.8%; *p* = 0.511), and lobar (10.9%–13.9%; *p* = 0.450) locations. BOMBS‐rated microbleeds similarly demonstrated stability in lobar (13.7%–18.8%; *p* = 0.226), basal ganglia (4.5%–6.0%; *p* = 0.512), and posterior fossa (3.4%–3.9%; *p* = 1.000) regions.

### 3.1 Bayesian Model Diagnostics

All Bayesian models satisfied the prespecified convergence criteria. No divergent transitions were observed. Across all monitored fixed‐effect, random‐effect, threshold, and variance parameters, the maximum rank‐normalized split‐$\hat{R}$ was 1.009, the minimum bulk and tail ESS values were 651 and 1271, respectively, and the minimum chain‐specific BFMI was 0.463. Model‐specific diagnostics and prior specifications are provided in Tables S3 and S4.

### 3.2 Temporal Trajectories

**FIGURE 2:**
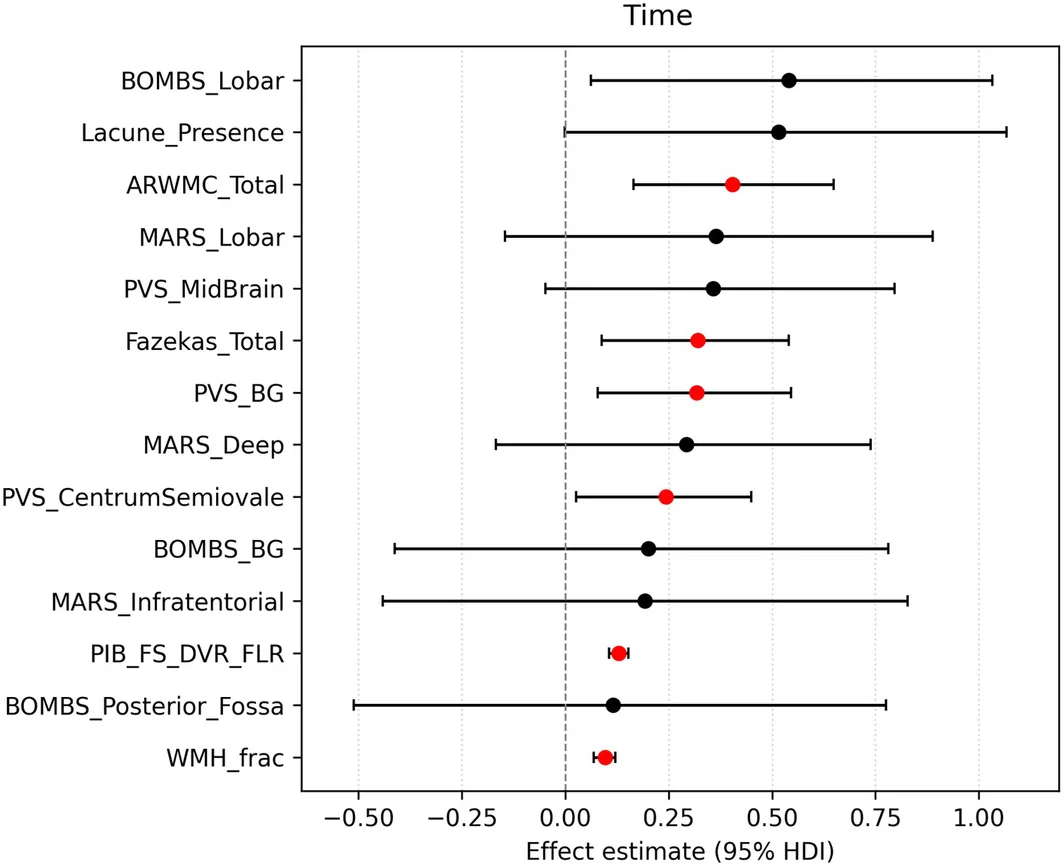
Forest plot illustrating the main effect of time (*z*‐score) on longitudinal trajectories of small vessel disease markers and amyloid PET burden across the study period. Estimates reflect the posterior mean beta with 95% highest density intervals (HDIs). ARWMC, Age‐Related White Matter Changes score; BG, basal ganglia; BOMBS, Brain Observer MicroBleed Scale; PIB_FS_DVR_FLR, Mean distribution volume ratio (DVR) of the tracer [^11^C] Pittsburgh Compound‐B (PIB) relative to whole‐cerebellum uptake across a FreeSurfer (FS)‐defined cortical composite (FLR) of frontal, lateral temporal, parietal, and retrosplenial cortices; MARS, Microbleed Anatomical Rating Scale; PVS, perivascular spaces; WMH_frac, white matter hyperintensity volume normalized to intracranial volume (WMH/ICV). Statistical significance: Red points indicate results with *p*‐corrected < 0.05. *p*‐corrected denotes the Benjamini–Hochberg FDR‐adjusted two‐sided posterior tail‐area *p*‐analogue.

Longitudinal neuroimaging assessment revealed significant progression across multiple metrics over the 3‐year interval (Figure 2). The following SVD markers showed increases: WMH/ICV exhibited a significant progression (*β* = 0.096, 95% HDI [0.069, 0.121], *p*_corrected_ < 0.001). ARWMC scores showed significant progression (*β* = 0.404, 95% HDI [0.165, 0.649], *p*_corrected_ = 0.004), as did Fazekas scores (*β* = 0.320, 95% HDI [0.088, 0.541], *p*_corrected_ = 0.010); amyloid‐β deposition also increased significantly (*β* = 0.130, 95% HDI [0.106, 0.152], *p*_corrected_ < 0.001). The BG‐ and Centrum Semiovale‐PVS score suggested significant progression (*β* = 0.318, 95% HDI [0.079, 0.546], *p*_corrected_ = 0.010 and *β* = 0.244, 95% HDI [0.026, 0.450], *p*_corrected_ = 0.025 respectively). No significant longitudinal progression was observed across the remaining SVD markers after applying Benjamini–Hochberg FDR correction.

### 3.3 Baseline ALPS Effects

**FIGURE 3:**
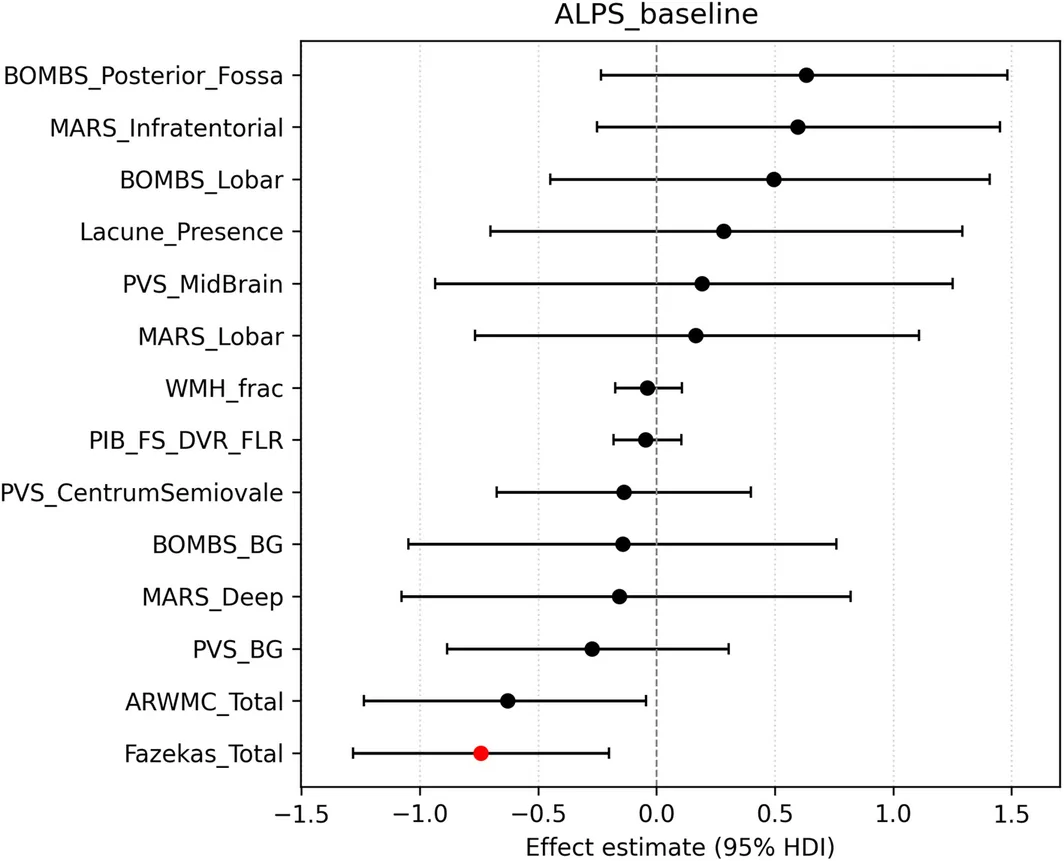
Forest plot depicting the cross‐sectional association of baseline DTI‐ALPS (*z*‐score) with small vessel disease markers and amyloid PET burden at baseline. Estimates reflect the posterior mean beta with 95% highest density intervals (HDIs). ALPS, diffusion tensor image analysis along the perivascular space; ARWMC, Age‐Related White Matter Changes score; BOMBS, Brain Observer MicroBleed Scale; BG, basal ganglia; PIB_FS_DVR_FLR, Mean distribution volume ratio (DVR) of the tracer [^11^C] Pittsburgh Compound‐B (PIB) relative to whole‐cerebellum uptake across a FreeSurfer (FS)‐defined cortical composite (FLR) of frontal, lateral temporal, parietal, and retrosplenial cortices; MARS, Microbleed Anatomical Rating Scale; PVS, perivascular spaces; WMH_frac, white matter hyperintensity volume normalized to intracranial volume (WMH/ICV). Statistical significance: Red points indicate results with *p*‐corrected < 0.05. p‐corrected denotes the Benjamini–Hochberg FDR‐adjusted two‐sided posterior tail‐area *p*‐analogue.

Cross‐sectional analysis examined the associations between baseline ALPS and SVD and amyloid markers (Figure 3). Higher baseline ALPS was significantly associated with a reduction in Fazekas score (*β* = −0.742, 95% HDI [−1.281, −0.201], *p*_corrected_ = 0.026). A similar trend was observed for ARWMC total score, but this did not remain significant after correction (*β* = −0.630, 95% HDI [−1.235, −0.044], *p*_corrected_ = 0.078). No significant relationships were observed for PVS measures, microbleed markers, lacune presence, amyloid burden, or WMH/ICV.

### 3.4 Effect of Baseline ALPS on Longitudinal Changes in SVD Burden, and Amyloid‐β Deposition

**FIGURE 4:**
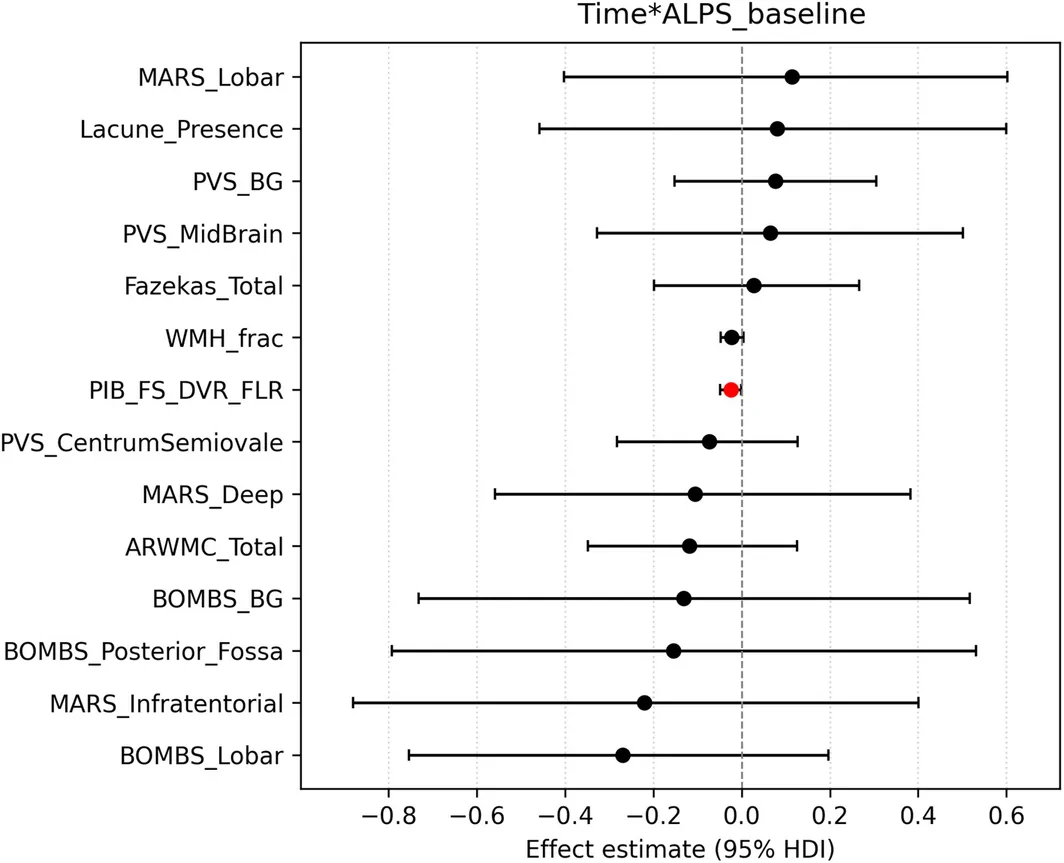
Forest plot showing the interaction between time (*z*‐score) and baseline DTI‐ALPS (*z*‐score) on longitudinal change in small vessel disease markers and amyloid PET burden. Estimates reflect posterior mean interaction coefficients with 95% highest density intervals (HDIs). ALPS, diffusion tensor image analysis along the perivascular space; ARWMC, Age‐Related White Matter Changes score; BG, basal ganglia; BOMBS, Brain Observer MicroBleed Scale; PIB_FS_DVR_FLR, Mean distribution volume ratio (DVR) of the tracer [^11^C] Pittsburgh Compound‐B (PIB) relative to whole‐cerebellum uptake across a FreeSurfer (FS)‐defined cortical composite (FLR) of frontal, lateral temporal, parietal, and retrosplenial cortices; MARS, Microbleed Anatomical Rating Scale; PVS, perivascular spaces; WMH_frac, white matter hyperintensity volume normalized to intracranial volume (WMH/ICV). Statistical significance: Red points indicate results with p‐corrected < 0.05. *p*‐corrected denotes the Benjamini–Hochberg FDR‐adjusted two‐sided posterior tail‐area *p*‐analogue.

**FIGURE 5:**
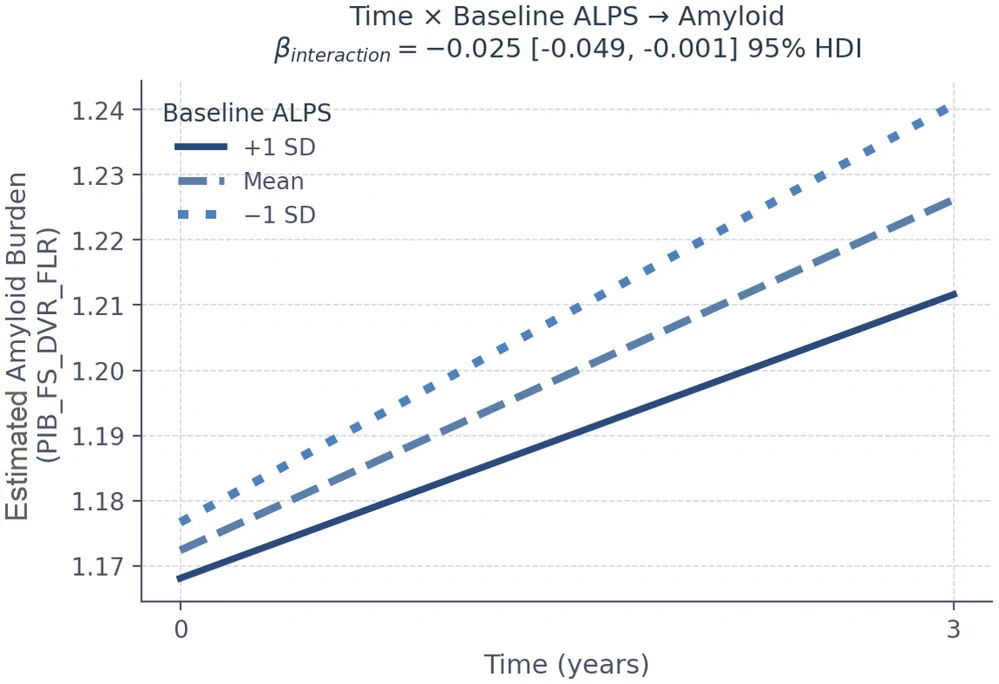
Estimated Amyloid Burden (PIB_FS_DVR_FLR) over time moderated by baseline ALPS. Plotted trajectories depict model‐estimated amyloid accumulation over a 3‐year interval for individuals at low (−1 SD, dotted), mean (dashed), and high (+1 SD, solid) baseline ALPS values. Trajectories are derived from a Bayesian Gaussian mixed‐effects model (adjusted for age, sex, education, and APOE4 status). PIB_FS_DVR_FLR, mean distribution volume ratio (DVR) of the tracer [^11^C] Pittsburgh Compound‐B (PIB) relative to whole‐cerebellum uptake across a FreeSurfer (FS)‐defined cortical composite (FLR) of frontal, lateral temporal, parietal, and retrosplenial cortices.

Assessment of the longitudinal influence of baseline ALPS, via the ALPS‐by‐time interaction term, revealed a significant finding (Figure 4). Higher baseline ALPS was associated with a reduction in the rate of amyloid accumulation over the 3‐year period (*β* = −0.025, 95% HDI [−0.049, −0.001], *p*_corrected_ = 0.039) (Figure 5). No significant ALPS‐by‐time interactions were observed for WMH/ICV (*β* = −0.023, 95% HDI [−0.047, 0.004], *p*_corrected_ = 0.077), ARWMC total score (*β* = −0.119, 95% HDI [−0.348, 0.126], *p*_corrected_ = 0.810), or Fazekas total score (*β* = 0.027, 95% HDI [−0.199, 0.266], *p*_corrected_ = 0.810). Baseline ALPS also did not significantly influence longitudinal change in PVS measures, lacune presence, microbleed markers, or other SVD outcomes.

### 3.5 Baseline DTI‐ALPS, SVD, and Amyloid‐β in Cognitive Outcomes

Higher baseline WMH/ICV was associated with poorer baseline PACC5 (*β* = −0.092, 95% HDI [−0.177, −0.011], *p*_corrected_ = 0.036), processing speed_z_ (*β* = −0.116, 95% HDI [−0.223, −0.010], *p*_corrected_ = 0.036), and executive function_z_ (*β* = −0.144, 95% HDI [−0.251, −0.029], *p*_corrected_ = 0.035). Higher baseline amyloid burden was associated with steeper PACC5 decline (*β* = −0.065, 95% HDI [−0.096, −0.033], *p*_corrected_ < 0.001). A positive temporal effect was observed for PACC5 (*β* = 0.040, 95% HDI [0.009, 0.071], *p*_corrected_ = 0.043), reflecting modest practice‐related improvement across the sample as a whole. No significant baseline or longitudinal associations were observed for baseline DTI‐ALPS, BG‐PVS/ICV, or CP/ICV after multiple‐comparison correction (Table 3).

**TABLE 3:** Associations between baseline glymphatic function, white matter hyperintensities, cerebral amyloid burden, and longitudinal trajectories of global cognition (PACC5), processing speed, and executive function.

	PACC5		Processing speed_z_		Executive function_z_	
	*β* (95% HDI)	*p*‐corrected	*β* (95% HDI)	*p*‐corrected	*β* (95% HDI)	*p*‐corrected
Temporal trajectories	0.040 (0.009, 0.071)	0.043*	−0.029 (−0.065, 0.008)	0.122	0.062 (−0.002, 0.130)	0.105
Baseline DTI‐ALPS	0.070 (−0.020, 0.156)	0.350	0.014 (−0.103, 0.131)	0.793	−0.020 (−0.137, 0.104)	0.793
Baseline WMH/ICV	−0.092 (−0.177, −0.011)	0.036*	−0.116 (−0.223, −0.010)	0.036*	−0.144 (−0.251, −0.029)	0.035*
Baseline BG_PVS/ICV	−0.005 (−0.080, 0.077)	0.894	0.046 (−0.052, 0.150)	0.767	0.035 (−0.072, 0.138)	0.767
Baseline CP/ICV	−0.053 (−0.137, 0.034)	0.561	0.000 (−0.112, 0.105)	0.983	−0.051 (−0.168, 0.062)	0.561
Baseline PIB_FS_DVR_FLR	−0.053 (−0.131, 0.021)	0.316	−0.027 (−0.123, 0.073)	0.603	0.065 (−0.036, 0.170)	0.316
Baseline DTI‐ALPS effect over time	0.025 (−0.013, 0.057)	0.500	−0.005 (−0.046, 0.036)	0.797	−0.017 (−0.088, 0.058)	0.797
Baseline WMH/ICV effect over time	−0.019 (−0.051, 0.014)	0.570	−0.012 (−0.050, 0.026)	0.570	0.019 (−0.049, 0.089)	0.570
Baseline BG_PVS/ICV effect over time	0.009 (−0.024, 0.041)	0.989	0.003 (−0.036, 0.040)	0.989	0.000 (−0.067, 0.071)	0.989
Baseline CP/ICV effect over time	−0.023 (−0.056, 0.012)	0.301	−0.045 (−0.084, −0.005)	0.071	−0.005 (−0.078, 0.066)	0.873
Baseline PIB_FS_DVR_FLR effect over time	−0.065 (−0.096, −0.033)	< 0.001*	−0.012 (−0.050, 0.023)	0.500	−0.027 (−0.095, 0.038)	0.500

*Note:* PACC5, Preclinical Alzheimer Cognitive Composite 5, computed as the mean of five cognitive component scores: Free and Cued Selective Reminding Test total recall, 0–96 scale (FCSRT96), delayed recall from the Logical Memory IIa subtest of the Wechsler Memory Scale (LMDR), Digit Symbol Substitution Test score (Digit Symbol), Mini‐Mental State Examination total score (MMSE), and Category Fluency total score (CAT; sum of animals, vegetables, and fruits); processing speed_z_: [z(Digit Symbol)‐z(TMT‐A)]/2; TMT‐A, Trail Making Test Part A; executive function_z_, −z[(TMT‐B)—(TMT‐A)]; TMT‐B, Trail Making Test Part B; DTI‐ALPS: diffusion tensor imaging–analysis along the perivascular space, WMH: white matter hyperintensities, BG‐PVS: basal ganglia perivascular spaces, CP: choroid plexus, ICV: intracranial volume, PIB_FS_DVR_FLR, mean [11C] Pittsburgh Compound‐B (PIB) distribution volume ratio (DVR), referenced to whole‐cerebellum uptake, across a FreeSurfer‐defined cortical composite comprising the frontal, lateral temporal, parietal, and retrosplenial cortices. Values are posterior mean regression coefficients (β) with 95% highest density intervals (HDI). All imaging markers and time were *z*‐score standardized prior to analysis. *p*‐corrected denotes the Benjamini–Hochberg FDR‐adjusted two‐sided posterior tail‐area *p*‐analogue. An asterisk (*) indicates statistically significant associations after correction (*p* < 0.05).

### 3.6 Choroid Plexus Segmentation Quality‐Control Sensitivity Analysis

Expert quality control was performed for 126 scans from 63 participants. Manual editing changed at least one voxel in 125 scans (99.2%), while one mask was unchanged. Automated and expert‐corrected masks demonstrated a mean Dice coefficient of 0.897 ± 0.085. Repeating the cognitive models using expert‐corrected CP/ICV did not identify significant associations between baseline CP/ICV and PACC5 (*β* = −0.110, 95% HDI [−0.265, 0.041], *p*_corrected_ = 0.476), processing speed (*β* = −0.083, 95% HDI [−0.315, 0.135], *p*_corrected_ = 0.677), or executive function (*β* = −0.038, 95% HDI [−0.223, 0.150], *p*_corrected_ = 0.677). Similarly, no significant CP/ICV‐by‐time interaction was observed for PACC5 (*β* = −0.005, 95% HDI [−0.069, 0.055], *p*_corrected_ = 0.886), processing speed (*β* = −0.064, 95% HDI [−0.138, 0.016], *p*_corrected_ = 0.341), or executive function (*β* = 0.009, 95% HDI [−0.120, 0.145], *p*_corrected_ = 0.886).

### 3.7 Crossing‐Fiber Sensitivity Analysis

After excluding voxels meeting the crossing‐fiber criterion, higher crossing‐fiber‐adjusted baseline ALPS remained significantly associated with a lower rate of longitudinal cortical amyloid accumulation (ALPS‐by‐time interaction: *β* = −0.028, 95% HDI [−0.052, −0.003], *p*_corrected_ = 0.022; Table S1). The estimate for Fazekas severity remained negative and directionally consistent with the primary analysis, although its magnitude was attenuated and it did not meet the significance criterion (*β* = −0.413, 95% HDI [−0.966, 0.181], *p*_corrected_ = 0.751). Findings for the remaining imaging outcomes were consistent with the primary analyses: no additional baseline associations were identified for SVD or amyloid measures (all *p*_corrected_ ≥ 0.255), and no significant ALPS‐by‐time interactions were observed for SVD progression (all *p*_corrected_ ≥ 0.254). Similarly, crossing‐fiber adjustment did not alter the cognitive findings, with no significant associations between baseline ALPS or its interaction with time and PACC5, processing speed, or executive function (all *p*_corrected_ ≥ 0.513; Table S2).

## 4 Discussion

In this longitudinal study of 204 cognitively normal older adults from the HABS, we investigated the association between the baseline DTI‐ALPS index and the progression of cerebral small vessel disease markers and amyloid deposition over a 3‐year follow‐up. Our analysis yielded four principal findings. First, SVD markers, including WMH/ICV and visual severity ratings, as well as cortical amyloid burden, showed significant progression over the study period. Second, the baseline ALPS index demonstrated a cross‐sectional association with lower Fazekas severity. Third, the only significant longitudinal interaction involved amyloid deposition, whereby higher baseline ALPS was associated with attenuated amyloid accumulation over time, while no significant moderating effect of ALPS on SVD progression was observed. Finally, analyses of DTI‐ALPS, CP/ICV, BG‐PVS/ICV, WMH/ICV volume, and amyloid‐β burden in relation to cognitive trajectories showed selective associations: greater baseline amyloid‐β burden was associated with steeper decline in global cognition (PACC5), whereas larger baseline WMH/ICV was associated with poorer baseline PACC5, processing speed, and executive function.

The observed 3‐year progression of WMH and visual SVD ratings in our cognitively normal cohort aligns with prior longitudinal studies demonstrating that SVD markers accumulate steadily with age, even in the absence of clinical cognitive impairment (Ide et al. 2024). WMH progression, quantified both volumetrically and on the Fazekas and ARWMC scales, remained the most robust longitudinal signal. The lack of significant longitudinal change in several binary SVD markers, such as lacunes and regional microbleed scores, likely reflects their relatively low prevalence and the limited sensitivity of binary measures to detect subtle changes over a 3‐year window in a healthy aging cohort.

DTI‐ALPS provides a valuable, non‐invasive window into perivascular water dynamics in periventricular white matter, capturing diffusion anisotropy along medullary veins where venous drainage and perivascular spaces converge and has shown consistent associations with aging and WMH volume (Taoka et al. 2024; Li et al. 2024). However, ALPS does not directly quantify glymphatic flux or global cerebrospinal fluid–interstitial fluid exchange and is influenced by white‐matter integrity, extracellular free water, and perivascular space dilation (Taoka et al. 2024). A higher ALPS‐index reflects predominant Brownian water diffusion in the radial (left–right) direction at the level of the lateral ventricular body. While the term ALPS‐index has become widely adopted and familiar within the field, it should be reported as increased or decreased, with its interpretation as a marker of glymphatic function remaining a matter for careful and context‐dependent consideration (Taoka et al. 2024).

In the present study, quantitative WMH/ICV, Fazekas scores, and ARWMC scores all exhibited significant longitudinal progression over the 3‐year follow‐up. However, these measures differed in their cross‐sectional relationships with baseline DTI‐ALPS: higher baseline ALPS was associated with lower Fazekas severity but not with quantitative WMH/ICV or ARWMC score. This likely reflects fundamental differences in what these metrics capture. First, the Fazekas scale encodes qualitative transitions in lesion morphology, from punctate lesions (Fazekas 1), to early confluence (Fazekas 2), to large confluent regions (Fazekas 3). Such transitions can occur with relatively modest increases in total WMH volume. For example, the emergence of new lesion confluence may substantially increase the Fazekas grade while producing only a small absolute volumetric change, thereby amplifying sensitivity to early structural reorganization. Second, in normal aging with limited WMH burden, early pathological changes may preferentially involve periventricular spatial patterns rather than global lesion expansion. These qualitative periventricular alterations, captured by both Fazekas scoring and DTI‐ALPS, may precede substantial volumetric accumulation. Prior work has shown that approximately 89% of deep WMH lesions are spatially connected to perivascular spaces, and that free water significantly mediates the association between PVS and WMH volume (*β* = 0.069, *p* = 0.037) (Huang et al. 2021). Together, these findings suggest that impaired perivascular clearance may contribute to interstitial fluid accumulation along specific perivascular pathways, driving WMH formation in spatially coherent patterns that are more readily detected by Fazekas grading than by bulk volumetric measures (Andere et al. 2022). Further studies are needed to clarify the validity and sensitivity of WMH volumetric measures in normal aging, particularly at lower Fazekas grades.

The greater longitudinal progression detected for the ARWMC score compared with the Fazekas score, alongside the stronger baseline association between DTI‐ALPS and Fazekas, reflects fundamental differences in the anatomical sensitivity of these scales (Sachdev et al. 2026; Wahlund et al. 2001; Kapeller et al. 2003). The ARWMC scale provides a granular regional assessment by scoring WMH severity across distinct anatomical territories, including frontal, parieto‐occipital, temporal, infratentorial, and basal ganglia, yielding a cumulative score that captures distributed lesion patterns and regional vulnerability effectively (Wahlund et al. 2001). In contrast, the stronger baseline association between DTI‐ALPS and Fazekas scores likely reflects their shared periventricular focus and underlying perivascular fluid dynamics, as DTI‐ALPS specifically probes diffusion along perivascular spaces in periventricular regions where cerebrospinal fluid influx and venous drainage are most prominent (Min et al. 2021). Aggregation of abnormalities across multiple territories by the ARWMC total score may therefore have reduced sensitivity to a localized periventricular association. Nevertheless, this finding should be interpreted cautiously because the baseline association between ALPS and Fazekas severity was attenuated after crossing‐fiber adjustment.

The attenuation of the Fazekas association after crossing‐fiber adjustment is methodologically plausible because the periventricular regions used for ALPS calculation contain complex configurations of projection, association, and subcortical fibers. Georgiopoulos et al. demonstrated that crossing fibers can inflate the apparent ALPS index and that avoiding regions with substantial crossing‐fiber contributions can alter observed between‐group differences (Georgiopoulos et al. 2024). Schilling et al. similarly showed that ALPS is influenced by radial diffusion asymmetry arising from local white‐matter geometry, including crossing fibers, fiber dispersion, and undulation (Schilling et al. 2025). Thus, attenuation of the Fazekas association suggests that the primary cross‐sectional finding was partly sensitive to the microstructural composition of the ALPS regions and should be interpreted cautiously. Nevertheless, the crossing‐fiber‐adjusted analysis should be viewed as a complementary, more restrictive evaluation rather than a replacement for the conventional ALPS calculation because it recalculates the index using a reduced set of voxels selected to minimize substantial crossing‐fiber contributions. It therefore evaluates the robustness of the findings to ROI voxel composition without constituting a definitive correction for all aspects of complex fiber architecture.

A principal finding of the study was the association between baseline ALPS and longitudinal amyloid accumulation. Participants with higher baseline ALPS demonstrated a slower rate of cortical amyloid deposition over 3 years. This association remained significant after excluding voxels with a high likelihood of substantial fiber crossing, indicating that it was not driven solely by crossing‐fiber contributions within the ALPS regions. This finding is biologically plausible given that the glymphatic system and related perivascular pathways have been implicated in interstitial amyloid‐β clearance (Simon et al. 2022). Consistent with this, a lower ALPS index, as an indirect marker of altered perivascular water dynamics, may be associated with greater amyloid‐beta burden through amyloid‐dependent pathways, although ALPS alone cannot determine whether impaired glymphatic transport is the specific mechanism involved (Zhang et al. 2025). Our results are concordant with prior cross‐sectional work demonstrating an inverse relationship between ALPS and amyloid burden in mild cognitive impairment and Alzheimer's disease, and extend these observations to the preclinical phase in cognitively normal individuals (Huang et al. 2024).

Our results further indicate that greater baseline amyloid‐β burden is associated with longitudinal decline in global cognition during normal aging. This finding is consistent with several longitudinal studies demonstrating accelerated cognitive decline in individuals with elevated amyloid burden (van der Kall et al. 2021; Donohue et al. 2017; Farrell et al. 2017). Prior evidence also shows that the risk of progression to mild cognitive impairment or dementia increases substantially with higher amyloid levels (van der Kall et al. 2021), and that widespread amyloid deposition is associated with greater cognitive decline than regionally confined accumulation (Ozlen et al. 2022). At the biological level, amyloid pathology has been linked to impaired synaptic plasticity, disrupted glutamatergic signaling, and reduced network efficiency, resulting in subtle but progressive deficits in cognitive processes that rely on coordinated large‐scale network function (Talantova et al. 2013; Elman et al. 2016). Multimodal imaging studies suggest that amyloid burden correlates more strongly with functional connectivity disruption and cerebral hypometabolism than with focal atrophy in early disease stages, providing a mechanistic basis for its association with composite cognitive decline rather than isolated domain‐specific impairment (Elman et al. 2016; Drzezga et al. 2011). The PACC5 was specifically designed to enhance sensitivity to amyloid‐related cognitive change by integrating measures of episodic memory, executive function, semantic processing, and global cognition (domains known to decline early in preclinical Alzheimer's disease) (Papp et al. 2017; Donohue et al. 2014). The observed longitudinal *β* estimate aligns with prior reports demonstrating steeper decline on PACC‐based composites among amyloid‐positive individuals, even in the presence of practice effects that may partially mask decline over time (Donohue et al. 2017; Mormino et al. 2017; Tort‐Merino et al. 2025).

Higher baseline WMH/ICV ratios were associated with poorer global cognition, processing speed, and executive function. WMH impairs cognition through a cascade of structural disconnection, secondary cortical atrophy, functional network disruption, and cortical hypometabolism (Kloppenborg et al. 2014; So et al. 2026). Specifically, WMH‐related demyelination and axonal injury disrupt cortico‐cortical and cortico‐subcortical transmission and may also provoke remote cortical thinning, demyelination, and iron loss in connected regions, indicating that the effects of WMHs extend beyond the visible lesion burden itself (Li et al. 2023). Furthermore, WMH involvement in the bilateral anterior thalamic radiation, forceps major, and inferior fronto‐occipital fasciculus is associated with domain‐specific impairment (Coenen et al. 2023). Regarding processing speed, evidence indicates tract‐specific disconnection, particularly within the forceps major, anterior thalamic radiation, and superior longitudinal fasciculus, accompanied by altered cortical thickness, notably in frontal regions (Coenen et al. 2023; Chen et al. 2023; da Silva et al. 2023). Executive function, in turn, appears to rely not only on these fronto‐subcortical disconnection pathways, including the inferior fronto‐occipital fasciculus, frontal aslant tract, and superior longitudinal fasciculus, but also on downstream metabolic and network‐level effects, including cortical hypometabolism in the frontal and posterior cingulate regions (So et al. 2026; Taghvaei et al. 2024). These findings support several plausible mechanisms underlying WMH‐related cognitive impairment, yet further studies are needed to identify more comprehensive mediators of WMH effects on global cognition, processing speed, and executive function.

The absence of significant ALPS‐by‐time interactions for SVD markers warrants discussion. Several factors may contribute to this null finding. The 3‐year follow‐up may be insufficient to capture the slow, cumulative effect of reduced perivascular water diffusivity on white matter and microvascular injury in a cognitively healthy population. Additionally, SVD progression is influenced by multiple vascular risk factors, including hypertension, diabetes, hyperlipidemia, and smoking, which may complicate efforts to isolate the independent contribution of perivascular fluid dynamics (Wang et al. 2021). It is also possible that the relationship between lower ALPS values and SVD is mediated indirectly through amyloid accumulation or neuroinflammation, rather than representing a direct pathway. Future studies with longer follow‐up intervals and more granular measures of vascular risk factor burden may be better positioned to detect such associations.

The following limitations should be acknowledged. First, the ALPS index is an indirect diffusion‐derived measure that has been proposed as a marker related to glymphatic function but whose specificity for glymphatic clearance remains debated. Fiber crossing, partial volume effects, and variability in ROI placement may influence ALPS estimates (Georgiopoulos et al. 2024), and validation against more direct measures of glymphatic clearance, such as intrathecal gadolinium‐enhanced MRI, remains limited. Second, the ALPS index was modeled as a baseline predictor, and thus the observed longitudinal relationships should be interpreted as associations rather than evidence of temporal precedence or prediction, and bidirectional or reverse‐causal interpretations cannot be excluded. Third, the primary models adjusted for age, sex, education, and APOE ϵ4 status, but vascular and cardiometabolic factors, including hypertension, antihypertensive use, diabetes, dyslipidemia, body mass index, smoking, and cardiovascular comorbidity, were not available for analysis. Because these factors may independently affect WMH burden, diffusion metrics, and amyloid deposition, residual confounding cannot be excluded. Fourth, the DTI acquisition used a relatively modest number of diffusion directions (35 volumes), which may affect tensor estimation accuracy. Fifth, the HABS cohort is predominantly white and highly educated, which may limit the generalizability of our findings to more diverse populations. Sixth, the 3‐year follow‐up, while providing meaningful longitudinal data, may be too short to capture the full impact of reduced perivascular diffusivity on SVD progression, particularly for markers with low prevalence or slow evolution such as lacunes and microbleeds. Seventh, independent assessments of attentional control, such as the Stroop color‐word interference task or antisaccade paradigms, were not feasible because these measures were not available in the dataset.

In conclusion, this study shows that in cognitively normal older adults, higher baseline ALPS was associated with lower Fazekas severity, although attenuation of this association after crossing‐fiber adjustment indicates sensitivity to the microstructural composition of the ALPS regions. In contrast, higher baseline ALPS was associated with slower short‐term cortical amyloid‐β accumulation, and this longitudinal association remained significant after crossing‐fiber adjustment. Furthermore, our findings indicate that baseline amyloid burden is associated with more rapid longitudinal cognitive decline, while greater baseline white matter hyperintensity volume is associated with poorer baseline global cognition, executive function, and processing speed. Collectively, these findings support ALPS as a candidate noninvasive diffusion marker of perivascular water dynamics, particularly in relation to longitudinal amyloid‐related change, while emphasizing that its relationship with SVD burden and its biological specificity require further validation. Future studies incorporating longer follow‐up and complementary measures of perivascular fluid transport are needed to clarify the vascular and amyloid‐related processes underlying these associations.

## Author Contributions

Conceptualization: N.A., I.K., and S.K. Study design: N.A., S.K. Data curation: N.A. Imaging data acquisition and quality control: N.A. and S.K. Data preprocessing and analysis: N.A., H.B., and Z.G. Statistical analysis: N.A. Writing – original draft: N.A., I.K., and D.S. Writing – review and editing: Z.G., H.B., K.F., S.K. Supervision: K.F., S.K. Final approval of the version to be published: All authors.

## Funding

The authors have nothing to report.

## Ethics Statement

This study is a secondary analysis of the de‐identified data from Harvard Aging Brain Study (HABS), a publicly available dataset and did not require additional ethical approval.

## Consent

Patient consent was not required for this study because it was a secondary analysis of the HABS dataset.

## Conflicts of Interest

The authors declare no conflicts of interest.

## Supporting information


**Table S1:** Posterior estimates from sensitivity analyses of neuroimaging outcomes using the crossing‐fiber‐adjusted DTI‐ALPS index.
**Table S2:** Posterior estimates from sensitivity analyses of cognitive outcomes using the crossing‐fiber‐adjusted DTI‐ALPS index.
**Table S3:** Prior specifications for the primary Bayesian models.
**Table S4:** Sampling and convergence diagnostics for the primary Bayesian models.

## Data Availability

The data that support the findings of this study are available from Harvard Aging Brain Study (HABS). Restrictions apply to the availability of these data, which were used under license for this study. Data are available from https://habs.mgh.harvard.edu with the permission of Harvard Aging Brain Study (HABS).
