# Supplementary material for: DTI‐ALPS as a Biomarker of Small Vessel Disease Progression and Amyloid‐β in Normal Aging: A 3‐Year Longitudinal Study
Source: Hum Brain Mapp. 2026 Sep 20;47(14):e70649. doi: 10.1002/hbm.70649 (PMC13590925; doi:10.1002/hbm.70649)
Supplement: Supplementary file 1 [file HBM-47-e70649-s001.docx]

This is the supplementary information of “DTI-ALPS as a Biomarker of Small Vessel Disease Progression and Amyloid-β in Normal Aging: A 3-Year Longitudinal Study” by Narges Azizi, Hoda Borooghani, Iman Kiani, Zeinab Gharaylou, Dina Seyedi, Kavous Firouznia, and Shahriar Kolahi*.

**Table of Contents**

**Table S1.** Posterior estimates from sensitivity analyses of neuroimaging outcomes using the crossing-fiber-adjusted DTI-ALPS index.

**Table S2.** Posterior estimates from sensitivity analyses of cognitive outcomes using the crossing-fiber-adjusted DTI-ALPS index.

**Table S3.** Prior specifications for the primary Bayesian models.

**Table S4.** Sampling and convergence diagnostics for the primary Bayesian models.

**Table S1. Posterior estimates from sensitivity analyses of neuroimaging outcomes using the crossing-fiber-adjusted DTI-ALPS index.**

| **Outcome** | **Time, β (95% HDI)** | **P-corrected** | **Baseline ALPS, β (95% HDI)** | **P-corrected** | **ALPS × time, β (95% HDI)** | **P-corrected** |
| --- | --- | --- | --- | --- | --- | --- |
| **WMH/ICV** | 0.100 (0.077, 0.127) | <0.001* | 0.048 (−0.093, 0.190) | 0.505 | −0.015 (−0.042, 0.010) | 0.254 |
| **Fazekas total score** | 0.348 (0.113, 0.592) | 0.009* | −0.413 (−0.966, 0.181) | 0.751 | −0.015 (−0.245, 0.220) | 0.903 |
| **ARWMC total score** | 0.433 (0.184, 0.687) | 0.005* | −0.319 (−0.992, 0.315) | 0.836 | −0.169 (−0.427, 0.071) | 0.845 |
| **Basal ganglia PVS rating** | 0.350 (0.103, 0.590) | 0.009* | 0.039 (−0.562, 0.682) | 0.887 | 0.095 (−0.142, 0.326) | 0.845 |
| **Centrum semiovale PVS rating** | 0.247 (0.036, 0.459) | 0.028* | 0.154 (−0.420, 0.658) | 0.867 | −0.071 (−0.280, 0.137) | 0.845 |
| **Midbrain PVS presence** | 0.394 (−0.041, 0.841) | 0.076 | 0.213 (−0.920, 1.346) | 0.867 | 0.025 (−0.453, 0.429) | 0.903 |
| **Lacune presence** | 0.517 (0.007, 1.054) | 0.157 | 0.905 (−0.036, 1.823) | 0.255 | 0.045 (−0.452, 0.582) | 0.869 |
| **MARS infratentorial** | 0.217 (−0.456, 0.852) | 0.604 | 0.622 (−0.242, 1.434) | 0.255 | −0.209 (−0.889, 0.453) | 0.835 |
| **MARS deep** | 0.303 (−0.169, 0.763) | 0.353 | −0.045 (−0.927, 0.937) | 0.940 | −0.192 (−0.653, 0.282) | 0.835 |
| **MARS lobar** | 0.364 (−0.132, 0.884) | 0.353 | 0.403 (−0.519, 1.352) | 0.549 | 0.115 (−0.413, 0.615) | 0.835 |
| **BOMBS lobar** | 0.590 (0.105, 1.107) | 0.100 | 0.676 (−0.275, 1.563) | 0.255 | −0.377 (−0.876, 0.103) | 0.826 |
| **BOMBS basal ganglia** | 0.213 (−0.375, 0.804) | 0.604 | −0.115 (−1.037, 0.764) | 0.935 | −0.116 (−0.725, 0.517) | 0.835 |
| **BOMBS posterior fossa** | 0.122 (−0.542, 0.795) | 0.717 | 0.663 (−0.232, 1.498) | 0.255 | −0.129 (−0.795, 0.542) | 0.835 |
| **Cortical amyloid burden** | 0.130 (0.107, 0.154) | <0.001* | −0.020 (−0.158, 0.132) | 0.800 | −0.028 (−0.052, −0.003) | 0.022* |

**Footnote:** Values are posterior mean β (95% HDI). Time and baseline ALPS were z-standardized. Continuous, ordinal, and binary outcomes were analyzed using Bayesian Gaussian, cumulative ordinal, and Bernoulli mixed-effects models, respectively. Models included subject-specific random intercepts and were adjusted for baseline age, sex, and education; the amyloid model was additionally adjusted for APOE4. p-corrected denotes the Benjamini–Hochberg FDR-adjusted two-sided posterior tail-area p-analogue. *p-corrected < 0.05. ALPS, Analysis Along the Perivascular Space; ARWMC, Age-Related White Matter Changes; BOMBS, Brain Observer MicroBleed Scale; HDI, highest density interval; ICV, intracranial volume; MARS, Microbleed Anatomical Rating Scale; PVS, perivascular spaces; WMH, white matter hyperintensities.

**Table S2. Posterior estimates from sensitivity analyses of cognitive outcomes using the crossing-fiber-adjusted DTI-ALPS index.**

| **Cognitive outcome** | **Time, β (95% HDI)** | **p-corrected** | **Baseline ALPS, β (95% HDI)** | **p-corrected** | **ALPS × time, β (95% HDI)** | **p-corrected** |
| --- | --- | --- | --- | --- | --- | --- |
| **PACC5** | 0.040 (0.006, 0.072) | 0.053 | 0.047 (−0.047, 0.136) | 0.937 | 0.022 (−0.011, 0.059) | 0.513 |
| **Processing speed** | −0.033 (−0.071, 0.005) | 0.098 | 0.001 (−0.120, 0.114) | 0.983 | −0.013 (−0.052, 0.026) | 0.513 |
| **Executive function** | 0.059 (−0.007, 0.132) | 0.098 | −0.026 (−0.142, 0.098) | 0.983 | −0.029 (−0.104, 0.049) | 0.513 |

**Footnote:** Values are posterior mean β (95% HDI). Time and baseline ALPS were z-standardized. Bayesian Gaussian mixed-effects models included subject-specific random intercepts and were adjusted for baseline age, sex, education, imaging predictors, and their interactions with time. p-corrected denotes the Benjamini–Hochberg FDR-adjusted two-sided posterior tail-area p-analogue. *p-corrected < 0.05. ALPS, Analysis Along the Perivascular Space; BG-PVS, basal ganglia perivascular spaces; CP, choroid plexus; HDI, highest density interval; ICV, intracranial volume; PACC5, Preclinical Alzheimer Cognitive Composite 5; WMH, white matter hyperintensities.

**Table S3. Prior specifications for the primary Bayesian models.**

| **Model class** | **Outcomes** | **Regression priors** | **Random-intercept prior** | **Residual/auxiliary priors** |
| --- | --- | --- | --- | --- |
| Gaussian imaging/amyloid | WMH/ICV; cortical amyloid | All coefficients, including intercept: $N\left( 0 , 2 \right)$ | $u_{i}=z_{i}\sigma_{u};\text{ }z_{i}\sim N\left( 0 , 1 \right);\text{ }\sigma_{u}\sim\mathrm{HalfNormal}\left( 1 \right)$ | $\sigma_{\epsilon}\sim\mathrm{HalfNormal}\left( 1 \right)$ |
| Gaussian cognition | PACC5; processing speed; executive function | All coefficients, including intercept: $N\left( 0 , 1.5 \right)$ | $u_{i}=z_{i}\sigma_{u};\text{ }z_{i}\sim N\left( 0 , 1 \right);\text{ }\sigma_{u}\sim\mathrm{HalfNormal}\left( 1 \right)$ | $\sigma_{\epsilon}\sim\mathrm{HalfNormal}\left( 1 \right)$ |
| Bernoulli logit | Midbrain PVS, lacunes, MARS, and BOMBS outcomes | Bambi autoscaled priors: intercept $N\left( 0 , 1.5 \right)$; time and ALPS $N\left( 0 , 1.000\text{–}1.004 \right)$; interaction $N\left( 0 , 0.997\text{–}1.002 \right)$; age $N\left( 0 , 0.164 \right)$; sex $N\left( 0 , 1 \right)$; education $N\left( 0 , 0.332\text{–}0.333 \right)$ | $u_{i}\sim N\left( 0 , \sigma_{u} \right)$; $\sigma_{u}\sim\mathrm{HalfNormal}\left( 13.417\text{–}13.426 \right)$ | Bernoulli likelihood with logit link |
| Cumulative logit | Fazekas, ARWMC, basal ganglia PVS, and centrum semiovale PVS | Bambi autoscaled priors: time and ALPS $N\left( 0 , 2.5 \right)$; interaction $N\left( 0 , 2.492 \right)$; age $N\left( 0 , 0.411 \right)$; sex $N\left( 0 , 5.072 \right)$; education $N\left( 0 , 0.832 \right)$ | $u_{i}\sim N\left( 0 , \sigma_{u} \right)$; $\sigma_{u}\sim\mathrm{HalfNormal}\left( 33.123 \right)$ | Ordered thresholds: $N\left( \left[ - , 2 , 02 \right] , 1 \right)$for three thresholds and $N\left( \left[ - , 2 , - , 0.670.672 \right] , 1 \right)$for four thresholds |

**Footnote:** $N\left( \mu, \sigma\right)$denotes a Normal distribution parameterized by mean and standard deviation. HalfNormal values denote scale parameters. Gaussian priors were explicitly specified in PyMC. Bernoulli and cumulative-logit priors were generated using Bambi 0.16.0’s default autoscaling procedure; small differences in scale reflect differences in the analytic samples and predictor distributions. ALPS, analysis along the perivascular space; ARWMC, Age-Related White Matter Changes; BOMBS, Brain Observer MicroBleed Scale; ICV, intracranial volume; MARS, Microbleed Anatomical Rating Scale; PACC5, Preclinical Alzheimer Cognitive Composite 5; PVS, perivascular spaces; WMH, white matter hyperintensities.

**Table S4. Sampling and convergence diagnostics for the primary Bayesian models.**

| **Outcome** | **Model** | **Retained draws/chain** | **Divergences** | **Minimum BFMI** | **Maximum** $\hat{R}$ | **Minimum bulk ESS** | **Minimum tail ESS** |
| --- | --- | --- | --- | --- | --- | --- | --- |
| WMH/ICV | Gaussian | 4,000 | 0 | 0.476 | 1.0076 | 707 | 1,561 |
| Cortical amyloid burden | Gaussian | 4,000 | 0 | 0.481 | 1.0088 | 651 | 1,271 |
| PACC5 | Gaussian | 2,000 | 0 | 0.463 | 1.0035 | 2,150 | 4,214 |
| Processing speed | Gaussian | 2,000 | 0 | 0.509 | 1.0025 | 1,818 | 2,759 |
| Executive function | Gaussian | 2,000 | 0 | 0.490 | 1.0029 | 2,301 | 3,444 |
| Fazekas total score | Cumulative logit | 2,000 | 0 | 0.549 | 1.0034 | 1,580 | 2,970 |
| ARWMC total score | Cumulative logit | 2,000 | 0 | 0.569 | 1.0034 | 1,603 | 2,669 |
| Basal ganglia PVS | Cumulative logit | 2,000 | 0 | 0.585 | 1.0027 | 1,535 | 2,918 |
| Centrum semiovale PVS | Cumulative logit | 2,000 | 0 | 0.578 | 1.0051 | 1,389 | 2,737 |
| Midbrain PVS | Bernoulli logit | 2,000 | 0 | 0.697 | 1.0027 | 2,098 | 3,640 |
| Lacune presence | Bernoulli logit | 2,000 | 0 | 0.826 | 1.0023 | 3,802 | 4,477 |
| MARS infratentorial | Bernoulli logit | 2,000 | 0 | 0.840 | 1.0030 | 5,081 | 4,663 |
| MARS deep | Bernoulli logit | 2,000 | 0 | 0.808 | 1.0027 | 3,273 | 4,183 |
| MARS lobar | Bernoulli logit | 2,000 | 0 | 0.819 | 1.0022 | 4,273 | 4,433 |
| BOMBS lobar | Bernoulli logit | 2,000 | 0 | 0.768 | 1.0024 | 2,912 | 4,439 |
| BOMBS basal ganglia | Bernoulli logit | 2,000 | 0 | 0.829 | 1.0029 | 5,142 | 4,732 |
| BOMBS posterior fossa | Bernoulli logit | 2,000 | 0 | 0.843 | 1.0043 | 5,697 | 4,807 |

**Footnote:** All models used four chains. Continuous imaging and amyloid models used 3,000 tuning iterations; all other models used 2,000. Values are the least favorable diagnostic across all monitored posterior parameters in each model, including fixed effects, subject effects, thresholds, and variance parameters. Prespecified criteria were rank-normalized split-$\hat{R}\leq1.01$, bulk and tail ESS ≥400, no divergent transitions, and BFMI ≥0.30. BFMI, Bayesian fraction of missing information; ESS, effective sample size; other abbreviations are defined in Tables S1 and S2.
